# Radiomics-based machine learning model for predicting secondary decompressive craniectomy in TBI patients after emergent craniotomy with bone flap replacement

**DOI:** 10.1186/s41016-025-00423-5

**Published:** 2026-01-08

**Authors:** Tiange Chen, Ganzhi Liu, Ziyuan Liu, Jiacheng Liu, Jinfang Liu, Zhongyi Sun

**Affiliations:** 1 Department of Neurosurgery, Xiangya Hospital, Central South University, No. 87 Xiangya Rd, Changsha, Hunan 410008 China; 2 Department of Neurosurgery, Xiangya School of Medicine, Central South University, Changsha, Hunan China

**Keywords:** Radiomics, Traumatic brain injury, Decompressive craniectomy, Machine learning

## Abstract

**Background:**

Secondary decompressive craniectomy (DC) is commonly integrated into tiered therapeutic protocols in the intensive care unit (ICU) to manage elevated intracranial pressure following traumatic brain injury (TBI). Identifying high-risk patients in advance could enable early intervention and help prevent further deterioration. This study aims to develop a machine learning-based predictive model using radiomics to assess the likelihood of secondary DC in TBI patients.

**Methods:**

A total of 65 patients were enrolled and divided into training and test cohorts through stratified random sampling with a 7:3 ratio. Radiomic features were extracted from pre-evacuation CT data. The most relevant features were identified through importance score computation, and various predictive models were assessed using distinct machine learning algorithms and data sources. Model performance was benchmarked to construct an optimal predictive model.

**Results:**

No statistically significant differences were observed in demographic and clinical characteristics between the DC and non-DC groups. The model based solely on demographic and clinical data did not achieve satisfactory performance, with an AUC below 0.5 in the test cohort. In radiomic modeling, the randomForest model demonstrated consistent performance, achieving an AUC of 0.83 in the test cohort. The multiomic model, which incorporated demographic, clinical, and radiomic features, showed improved predictive performance, with the cforest model achieving an AUC of 0.87 in the training cohort and 0.86 in the test cohort.

**Conclusion:**

We developed radiomics-based predictive models to assess the likelihood of progressively refractory intracranial hypertension leading to secondary DC in a selected cohort of TBI patients who had undergone emergent craniotomy for hematoma evacuation with bone flap replacement. The model relying solely on radiomic features extracted from the lesion demonstrated satisfactory performance. When these features were integrated with demographic and clinical data to create a multiomic model, predictive performance further improved. These findings highlight the model’s potential to identify high-risk patients, enabling early intervention to prevent further deterioration.

**Supplementary Information:**

The online version contains supplementary material available at 10.1186/s41016-025-00423-5.

## Background

Intracranial hypertension, a recognized sequela of Traumatic Brain Injury (TBI), poses a consequential risk for secondary injury, leading to adverse TBI outcomes [1, 2]. Although medical interventions often effectively manage elevated intracranial pressure (ICP), there are instances where patients experience progressive deterioration despite maximal therapeutic interventions. In such cases, decompressive craniectomy (DC) emerges as a potential recourse. DC offers a viable avenue for accommodating cerebral edema, effectively alleviating mass effects on vital brain structures, and enhancing cerebral autoregulation and blood flow [3]. The TBI research community has witnessed substantial efforts to address various aspects of DC, including indications, timing, and surgical techniques in different patient populations. This is evidenced by the conduct of several randomized trials [4–7]. These studies delineate favorable outcomes in DC-treated patients, including enhanced ICP control, reduced episodes of intracranial hypertension, and the potential for improved functional and quality-of-life measures compared to those managed solely with medical interventions.

Secondary DC is typically performed as a life-saving measure in cases of refractory intracranial hypertension in the intensive care unit (ICU). Unlike the decision-making process for primary DC, which is generally guided by established consensus guidelines [3, 8], the choice to pursue secondary DC remains a complex and debated issue among clinicians and researchers in the field of TBI. This is especially true for patients who undergo emergent craniotomy for hematoma evacuation and bone flap replacement, only to later experience refractory intracranial hypertension requiring secondary DC. While many surgeons currently rely on real-time ICP measurements to guide the decision for secondary DC, these measurements reflect only the immediate ICP status [9]. By the time sustained refractory ICP elevation is observed, it may already be too late for timely intervention. Identifying patients at risk for this progression and providing early intervention to prevent it remains an unresolved challenge.

Radiomics, an emerging technology in medical imaging, provides a wealth of quantitative, high-throughput data derived from radiographic images [10]. It enables comprehensive, automated analysis of various phenotypic features, such as shape and texture, which may reflect the underlying biological properties of lesions through artificial intelligence (AI) techniques. While radiomics has increasingly been used to develop noninvasive imaging-based biomarkers for clinical decision support in oncology, its application in TBI remains quite limited. Only a handful of studies have explored its potential for predicting hematoma progression [11, 12], in-hospital mortality [13], and long-term outcomes [14]. However, to our knowledge, no studies have applied radiomics to predict the likelihood of progressively refractory intracranial hypertension requiring secondary DC.

In our study, we focused on developing a predictive radiomics-based model to assess the likelihood of progressively refractory intracranial hypertension leading to secondary DC. We specifically enrolled TBI patients who underwent emergent craniotomy for hematoma evacuation with bone flap replacement in the ICU, some of whom later developed refractory intracranial hypertension, necessitating secondary DC. This patient group provided a unique opportunity to explore this issue. Radiomic features were extracted from pre-evacuation CT data, and the most relevant features were identified based on their Gini importance scores. We then evaluated various predictive models using different machine learning algorithms to predict the likelihood of secondary DC due to refractory intracranial hypertension.

## Methods

### Patients

**Fig. 1:**
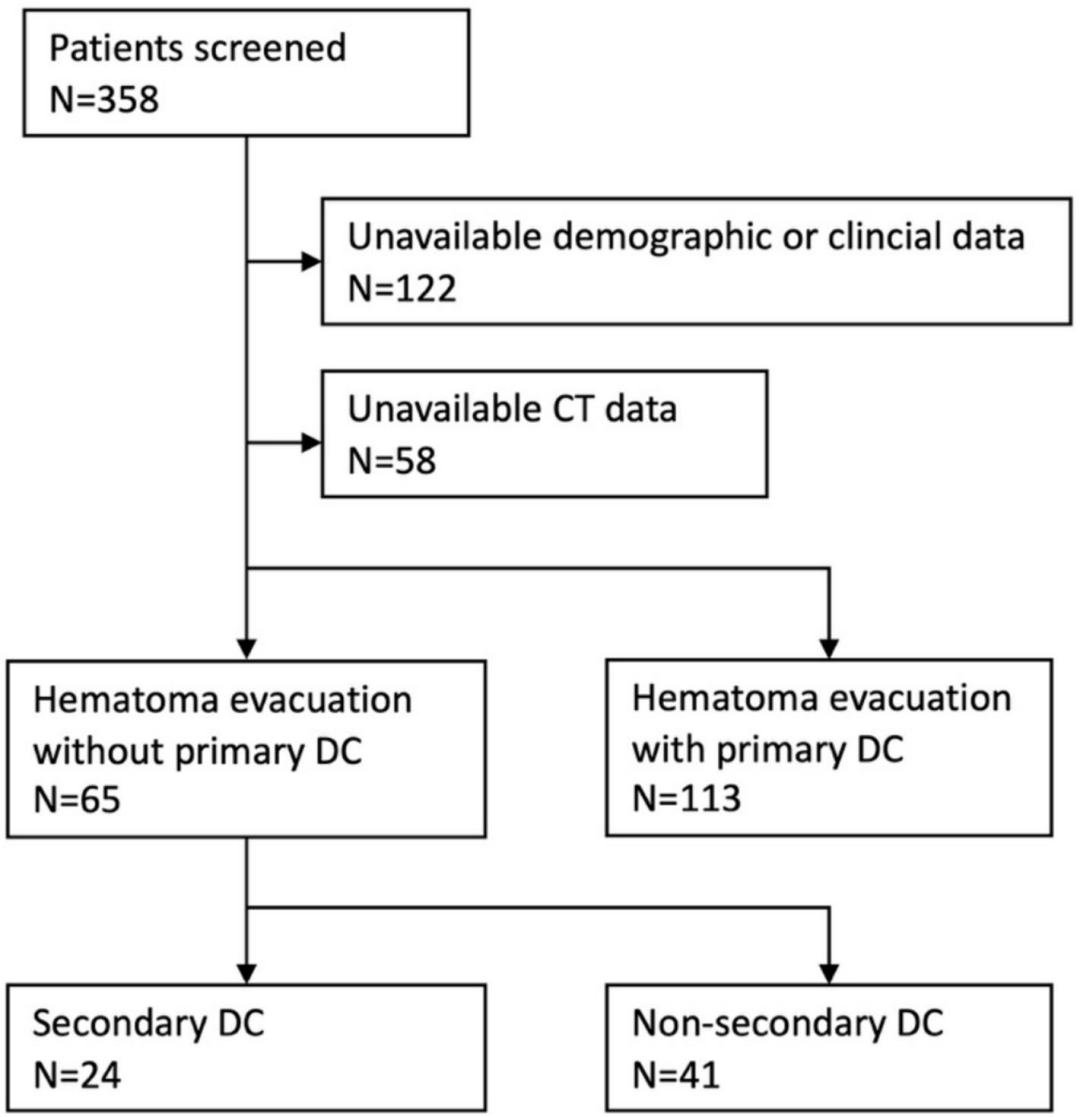
Patient inclusion flow chart. A total of 358 consecutive patients with acute severe TBI were initially screened. Sixty-five patients met the inclusion criteria and were included in the analysis, of whom 24 underwent secondary DC and 41 did not. DC, decompressive craniectomy; TBI, traumatic brain injury

This single-center retrospective study included patients aged 18–70 with acute severe TBI who underwent emergent craniotomy for hematoma evacuation and bone flap replacement at our medical center between 2018 and 2022. Additional inclusion criteria encompassed patients who underwent lateral ventricular cannulation for ICP monitoring and had available CT data before hematoma evacuation. Exclusion criteria included pregnancy, a history of substance or alcohol misuse, and previous brain surgical interventions. A total of 65 patients were included, with 24 undergoing secondary DC and 41 not undergoing DC, forming the DC group and non-DC group (NDC group), respectively (as shown in Fig. 1).

### Demographic and clinical characteristics

All data were obtained from the patients’ medical records utilizing the electronic database. Demographic characteristics, including sex, age, date of injury, and mechanism of injury, were recorded upon admission to the hospital. Clinical profiles, such as GCS score and pupil light reflex status, were documented both upon admission and within 8 h after the emergent evacuation procedure. Furthermore, data regarding ICP monitoring, CT Rotterdam score, and relevant blood laboratory tests, such as hemoglobin (HB), white blood cell count (WBC), and albumin (ALB), were collected within 8 h after the emergent evacuation procedure.

### Image acquisition and radiomic features extraction

All CT scans were performed using a 64-slice multidetector CT system (Siemens, Erlanger, Germany). The specific parameters employed were as follows: tube voltage set at 120 kV, tube current ranging from 160 to 250 mA, and matrix size of 512 × 512. The section thickness was set at 1 mm. The pre-evacuation CT scan was performed prior to the surgical procedure, typically within 2 h.

Segmentation of the region of interest (ROI) was performed using the open-source software 3D-Slicer (version 5.2.2). The ROI encompasses the volume of both intracranial hemorrhage and its peripheral edema in three dimensions, with only the largest lesion addressed in the case of multiple occurrences. The pyradiomics package (version 3.0.1), based on Python, was employed to extract image features from the segmented ROI. A total of 112 radiomic features were extracted from each ROI for every patient. These features comprised 5 diagnostic features and 107 original features, of which the latter were further categorized into seven groups: shape-based features, first-order statistics, gray level co-occurrence matrix (GLCM) features, gray level dependence matrix (GLDM) features, gray level run length matrix (GLRLM) features, gray level size zone matrix (GLSZM) features, and neighboring gray tone difference matrix (NGTDM) features [15].

### Radiomic feature selection

We utilized feature importance filter to discern significant features based on the model generated by a chosen machine learning approach [16, 17]. Patient data in our study underwent stratified random sampling, dividing it into a training cohort and a test cohort with a ratio of 7:3. Within the training cohort, we employed the ranger algorithm to compute the Gini importance score, also known as the Mean Decrease in Impurity (MDI). This score evaluates the importance of each feature by summing the number of splits across all trees that include the feature, weighted by the number of samples it splits [18]. The features were then ranked in descending order, with a higher Gini score indicating greater relevance.

### Machine learning model

The predictive modeling utilized six distinct machine learning algorithms: ctree [19, 20], cforest [19], gbm [21], kknn [22], randomForest [23], and svm [24]. Detailed information regarding each algorithm and their corresponding hyperparameters is available in Supplementary Table 1. To ensure the robustness and reliability of the training results, model construction and parameter tuning were performed exclusively within the training set. Specifically, leave-one-out cross-validation (LOO-CV) was applied within the training set to optimize model performance and select features, ensuring that the test data were not involved at any stage of model training or feature selection. The final model derived from the training phase was then independently evaluated on the test set to assess its generalization performance. Model evaluation was based on the primary performance metric, the area under the receiver operating characteristic curve (AUC), with accuracy (ACC) used as a supplementary metric. Both metrics were computed for the LOO-CV and test cohorts.

### Statistical analysis

Statistical analysis was conducted using GraphPad software (version 9, GraphPad Inc., USA). The continuous variables were subjected to either the Mann–Whitney test or *t*-test, while proportions were compared using the Fisher exact test or Chi-square test, to discern the differences between the DC group and NDC group. The *p*-value was adjusted for false discovery rate (FDR) using the Benjamini–Hochberg method, with statistical significance set at *p* < 0.05 (two-sided). To benchmark machine learning models, the R package mlr3 was utilized as a framework, and all experiments were primarily coded in R (version 4.3.0). To assess segmentation reproducibility, a subset of 10 cases was randomly selected, and the ROIs were manually segmented by two experienced neurosurgeons. The Dice Similarity Coefficient (DSC) was calculated for spatial overlap on the CT slice showing the maximum hematoma area, with a mean DSC of 0.79 ± 0.04, indicating good agreement between raters.

## Results

### Demographic and clinical characteristics

The data from 65 patients were stratified through random sampling into a training cohort (*n* = 46) and a test cohort (*n* = 19), employing a 7:3 ratio. Table 1 presents the demographic and clinical characteristics of patients in the DC and NDC groups. No statistically significant differences in demographic and clinical characteristics were observed between the two groups across the total, training, and test cohorts.

**Table 1:** Comparison of demographic and clinical characteristics

	**Total (*n* = 65)**			**Training Cohort (*n* = 46)**			**Test Cohort (*n* = 19)**		
**Characteristics**	**NDC group**	**DC group**	**Adjusted* p***	**NDC group**	**DC group**	**Adjusted *p***	**NDC group**	**DC group**	**Adjusted* p***
Number of patients	41	24		29	17		12	7	
Gender			0.37			0.55			> 0.99
Male	32 (78.0%)	22 (91.6%)		21 (72.4%)	15 (88.2%)		11 (91.6%)	7 (100%)	
Female	9 (22.0%)	2 (8.4%)		8 (27.6%)	2 (11.8%)		1 (8.4%)	0	
Age (years)	48.5 ± 14.8	55.1 ± 10.2	0.37	46.9 ± 14.5	55.5 ± 11.2	0.30	52.4 ± 15.5	54.4 ± 7.7	0.95
Mechanisms of injury			0.71			0.57			0.95
Traffic accident	20 (48.8%)	13 (54.2%)		18 (62.1%)	11 (64.7%)		2 (16.7%)	2 (28.6%)	
Falling	17 (41.5%)	11 (45.8%)		9 (31.0%)	6 (35.3%)		8 (66.7%)	5 (71.4%)	
Assault	1 (2.4%)	0		0	0		1 (8.3%)	0	
Others	3 (7.3%)	0		2 (6.9%)	0		1 (8.3%)	0	
Adm.LPD	2.35 ± 0.44	2.39 ± 0.95	0.94	2.38 ± 0.44	2.38 ± 0.86	> 0.99	2.29 ± 0.45	2.43 ± 1.24	0.93
Adm.LPRFX			0.56			0.57			0.86
Positive	39 (95.1%)	21 (87.5%)		27 (93.1%)	15 (88.2%)		12 (100%)	6 (85.7%)	
Negative	2 (4.9%)	3 (12.5%)		2 (6.9%)	2 (11.8%)		0	1 (14.3%)	
Adm.RPD	2.36 ± 0.43	2.68 ± 1.04	0.37	2.38 ± 0.44	2.65 ± 0.88	0.63	2.29 ± 0.45	2.79 ± 1.44	0.94
Adm.RPRFX			0.42			0.55			0.81
Positive	39 (95.1%)	20 (83.3%)		27 (93.1%)	14 (82.4%)		12 (100%)	6 (85.7%)	
Negative	2 (4.9%)	4 (16.7%)		2 (6.9%)	3 (17.6%)		0	1 (14.3%)	
Adm.PRFXscore	1.1 ± 0.4	1.3 ± 0.7	0.45	1.1 ± 0.5	1.3 ± 0.7	0.54	1.0 ± 0.1	1.3 ± 0.8	0.93
Adm.GCS	10.9 ± 3.1	10.2 ± 2.7	0.54	11.4 ± 2.9	10.3 ± 3.0	0.54	9.6 ± 3.0	10.0 ± 2.0	0.93
Adm.GCS-E	2.7 ± 0.9	2.4 ± 0.8	0.39	2.8 ± 0.9	2.4 ± 0.9	0.58	2.3 ± 0.9	2.3 ± 0.5	> 0.99
Adm.GCS-V	3.2 ± 1.5	2.9 ± 1.5	0.57	3.6 ± 1.4	2.9 ± 1.6	0.66	2.4 ± 1.5	2.7 ± 1.1	0.94
Adm.GCS-M	5.0 ± 1.0	4.9 ± 0.8	0.91	5.0 ± 1.0	4.9 ± 0.9	0.91	4.9 ± 1.1	5.0 ± 0.6	0.95
ICP	12.9 ± 6.8	14.1 ± 5.6	0.68	10.9 ± 5.2	14.1 ± 6.0	0.69	17.7 ± 8.3	14.1 ± 4.9	0.91
Rotterdam score	3.6 ± 1.1	4.0 ± 0.9	0.43	3.5 ± 1.0	4.0 ± 0.8	0.56	4.0 ± 1.4	4.1 ± 1.2	0.95
Post.LPD	1.83 ± 0.33	1.69 ± 0.32	0.40	1.84 ± 0.36	1.74 ± 0.26	0.59	1.79 ± 0.26	1.57 ± 0.45	> 0.99
Post.LPRFX			0.30			0.10			> 0.99
Positive	36 (87.8%)	16 (66.7%)		27 (93.1%)	10 (58.9%)		9 (75.0%)	6 (85.7%)	
Negative	5 (12.2%)	8 (33.3%)		2 (6.9%)	7 (41.1%)		3 (25.0%)	1 (14.3%)	
Post.RPD	1.81 ± 0.35	1.77 ± 0.46	0.90	1.83 ± 0.38	1.82 ± 0.50	> 0.99	1.79 ± 0.26	1.64 ± 0.38	0.85
Post.RPRFX			0.37			0.06			0.99
Positive	36 (87.8%)	15 (62.5%)		27 (93.1%)	9 (53.0%)		9 (75.0%)	6 (85.7%)	
Negative	5 (12.2%)	9 (37.5%)		2 (6.9%)	8 (47.0%)		3 (25.0%)	1 (14.3%)	
Post.PRFXscore	1.2 ± 0.7	1.7 ± 0.9	0.37	1.1 ± 0.5	1.8 ± 0.9	0.07	1.5 ± 0.9	1.3 ± 0.8	0.94
Post.GCS	10.5 ± 3.5	8.7 ± 3.2	0.25	10.7 ± 3.5	9.3 ± 3.2	0.99	10.1 ± 3.8	7.3 ± 3.2	> 0.99
Post.GCS-E	2.4 ± 1.0	2.0 ± 0.9	0.48	2.4 ± 1.0	2.1 ± 0.9	0.52	2.3 ± 1.1	1.7 ± 0.9	0.86
Post.GCS-V	3.0 ± 1.8	2.2 ± 1.5	0.32	3.0 ± 1.8	2.4 ± 1.6	0.46	2.8 ± 1.7	1.7 ± 1.1	> 0.99
Post.GCS-M	5.1 ± 0.9	4.5 ± 1.3	0.28	5.2 ± 0.7	4.8 ± 1.1	0.57	5.0 ± 1.3	3.9 ± 1.5	> 0.99
Hemoglobin	131.8 ± 19.6	126.4 ± 15.3	0.46	132.3 ± 18.6	126.7 ± 12.6	0.56	130.6 ± 22.7	125.5 ± 21.7	0.95
White blood cell	14.9 ± 5.3	14.7 ± 4.6	0.91	14.7 ± 6.1	15.1 ± 4.1	0.93	15.7 ± 2.9	13.7 ± 5.8	0.84
Neutrophils	85.4 ± 5.6	85.9 ± 4.1	0.86	85.0 ± 6.5	86.3 ± 2.8	0.58	86.4 ± 2.3	85.0 ± 6.5	0.88
Lymphocytes	7.7 ± 4.3	7.5 ± 3.3	0.91	8.3 ± 4.9	7.2 ± 2.4	0.55	6.5 ± 1.7	8.4 ± 4.9	0.99
Platelet	185.3 ± 58.7	164.6 ± 36.2	0.44	189.7 ± 61.2	167.9 ± 38.2	0.50	174.7 ± 53.6	156.8 ± 32.3	0.81
PT	13.7 ± 1.1	13.9 ± 1.3	0.67	13.6 ± 1.1	14.1 ± 1.5	0.47	14.0 ± 0.7	13.5 ± 0.9	0.93
APTT	30.7 ± 3.5	30.3 ± 3.8	0.85	30.6 ± 3.4	30.8 ± 4.4	0.96	30.7 ± 3.9	28.9 ± 1.6	> 0.99
INR	1.1 ± 0.1	1.1 ± 0.1	0.75	1.1 ± 0.1	1.1 ± 0.1	0.56	1.1 ± 0.1	1.1 ± 0.1	> 0.99
Fasting blood glucose	2.8 ± 0.9	2.5 ± 0.8	0.42	2.9 ± 1.1	2.6 ± 0.9	0.55	2.7 ± 0.6	2.3 ± 0.4	> 0.99
Albumin	43.4 ± 3.5	43.1 ± 4.0	0.88	43.1 ± 3.5	42.6 ± 4.0	0.86	44.1 ± 3.5	44.6 ± 3.9	0.94
Potassium	3.6 ± 0.4	3.5 ± 0.3	0.49	3.6 ± 0.3	3.5 ± 0.3	0.54	3.7 ± 0.5	3.6 ± 0.4	0.98
Sodium	140.0 ± 3.4	141.4 ± 4.8	0.37	140.0 ± 3.5	141.2 ± 4.7	0.56	139.9 ± 3.5	141.8 ± 5.4	0.80
Chloride	102.5 ± 2.7	102.6 ± 3.7	0.88	102.6 ± 2.4	102.4 ± 3.8	0.95	102.5 ± 3.4	103.4 ± 3.8	0.95
Disch.GCS	12.8 ± 2.5	10.8 ± 3.7	0.37	13.0 ± 2.5	10.6 ± 3.4	0.08	12.5 ± 2.5	11.1 ± 4.6	0.80

*DC* decompressive craniectomy, *NDC *non-decompressive craniectomy, *Adm *upon administration to the hospital, *LPD *left pupil diameter, *LPRFX *left pupil light reflex, *RPD *right pupil diameter, *RPRFX *right pupil light reflex, *PRFXscore *pupil light reflex score, defined by assigning values of 1 for bilateral reflex positive, 2 for unilateral reflex negative, and 3 for bilateral reflex negative, *GCS *Glasgow Coma Scale, *ICP *intracranial pressure, *Post *post emergent evacuation procedure within 8 h, *PT *prothrombin time, *APTT *activated partial thromboplastin time, *INR *international normalized ratio, *Disch* discharged from the hospital

### Radiomic modeling utilizing pre-evacuation CT data

**Fig. 2:**
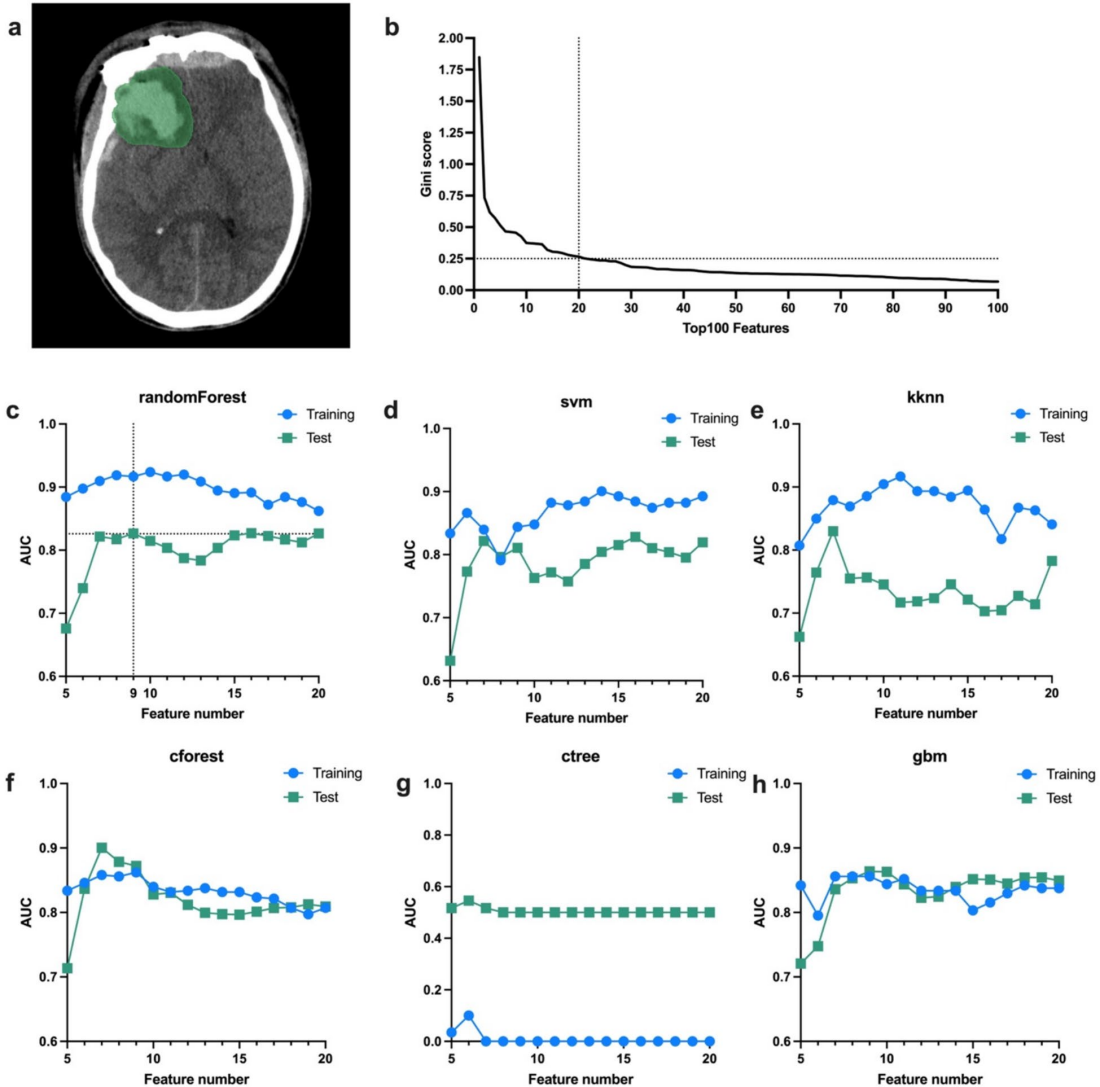
Radiomic modeling utilizing pre-evacuation CT data.** a** Representative ROI, delineated layer by layer, encompassing intracranial hemorrhage and surrounding edema. **b** Gini scores ranked in descending order, with all top 20 features having scores greater than 0.25. **c**–**h** Effect of feature number on the AUC for each machine learning algorithm. **c** The randomForest model demonstrated robustness, with higher AUC in both the training and test cohorts. Using the top 9 features, it achieved an AUC of 0.92 in the training cohort and 0.83 in the test cohort. ROI, region of interest; AUC, area under the curve

The ROI encompassing intracranial hemorrhage and surrounding edema was carefully delineated layer by layer on the pre-evacuation CT scan, as shown in Fig. 2a. In the training cohort, radiomic features were ranked by Gini score, with the feature Maskoriginal_VolumeNum topping the list at a score of 1.85. The top 20 features all had Gini scores greater than 0.25, as shown in Fig. 2b and detailed in Supplementary Table 2. The top features, ranging from the top 5 to 20, were then used as inputs for various machine learning algorithms. Different feature sets led to variations in the AUC, illustrating how the number of features impacted algorithm performance, as shown in Fig. 2c–h. Notably, the randomForest model (Fig. 2c) demonstrated consistent performance, achieving an AUC of 0.92 in the training cohort and 0.83 in the test cohort when using the top 9 features, with a sensitivity of 0.75 and specificity of 0.86. The corresponding ACC results for these models are shown in Supplementary Fig. 1, and the SHAP (SHapley Additive exPlanations) values for each feature in the radiomic model are presented in Supplementary Fig. 2.

### Predictive modeling utilizing demographic and clinical data

Due to collinearity concerns, several demographic and clinical features were excluded from the analysis, including the GCS-E, GCS-V, GCS-M, and pupil light reflex score (PRFXscore). This resulted in a final set of 28 features: 22 continuous variables and 6 categorical variables.

**Fig. 3:**
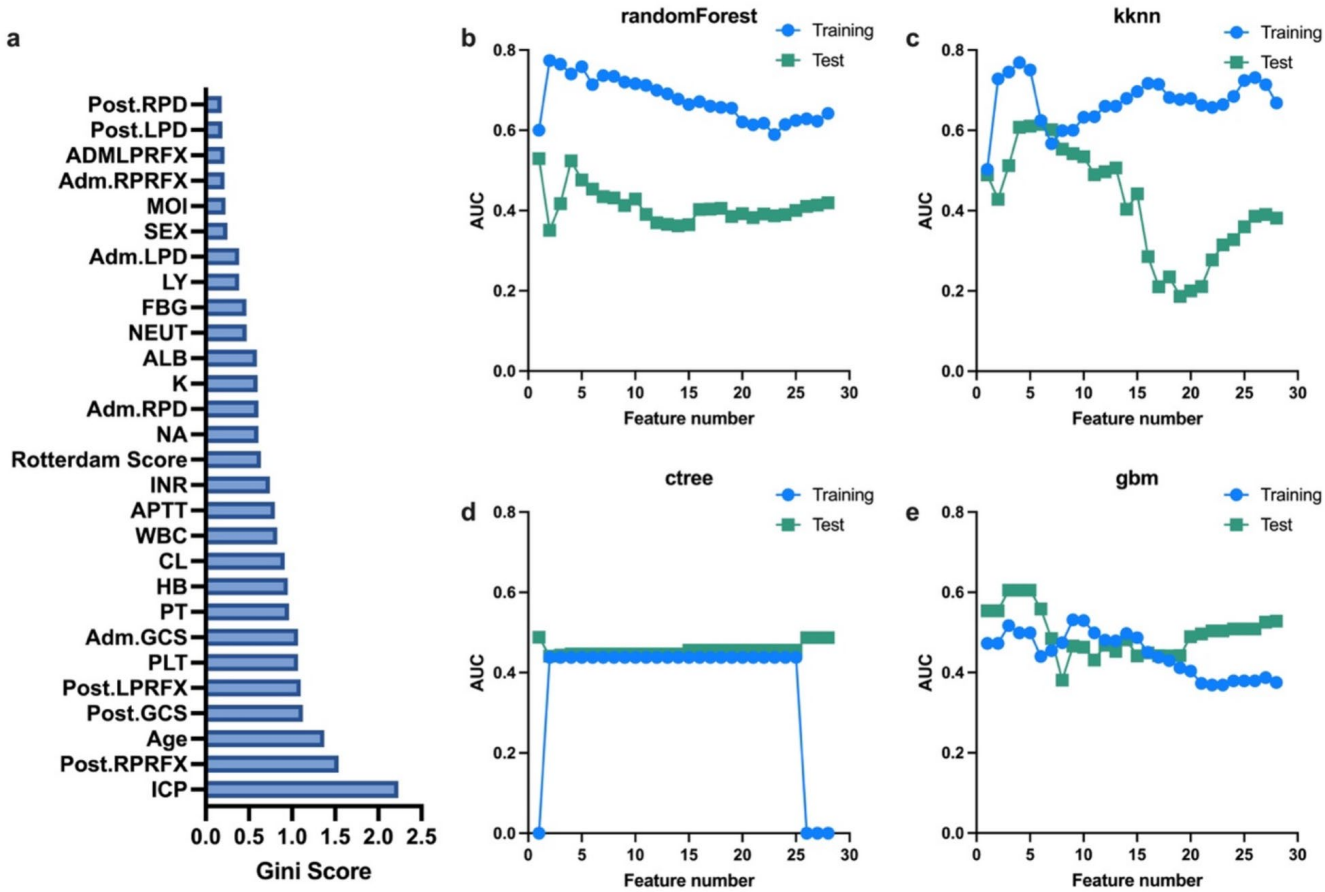
Predictive modeling utilizing demographic and clinical data**. a** Features ranked by Gini score. **b**–**e** The impact of varying feature numbers on AUC for each machine learning algorithm. The average AUC for each model remained below 0.5: 0.41 ± 0.04 for randomForest (**b**), 0.41 ± 0.14 for kknn (**c**), 0.46 ± 0.01 for ctree (**d**), and 0.49 ± 0.06 for gbm (**e**). AUC, area under the curve

In the training cohort, features were ranked by Gini score (Fig. 3a), and top 1 to 28 features were integrated as inputs for the machine learning algorithms. The svm model, which could not handle factor types, and the cforest model, which is not suitable for high-level factor types, were excluded from the analysis. Each algorithm’s performance was evaluated based on the number of features included. Despite this, the average AUC for all models remained suboptimal in the test cohort, with values below 0.5 (randomForest: 0.41 ± 0.04, kknn: 0.41 ± 0.14, ctree: 0.46 ± 0.01, and gbm: 0.49 ± 0.06), as shown in Fig. 3b–e. The ACC results for these models are detailed in Supplementary Fig. 3.

### Combining demographic, clinical, and CT data for multiomic model

**Fig. 4:**
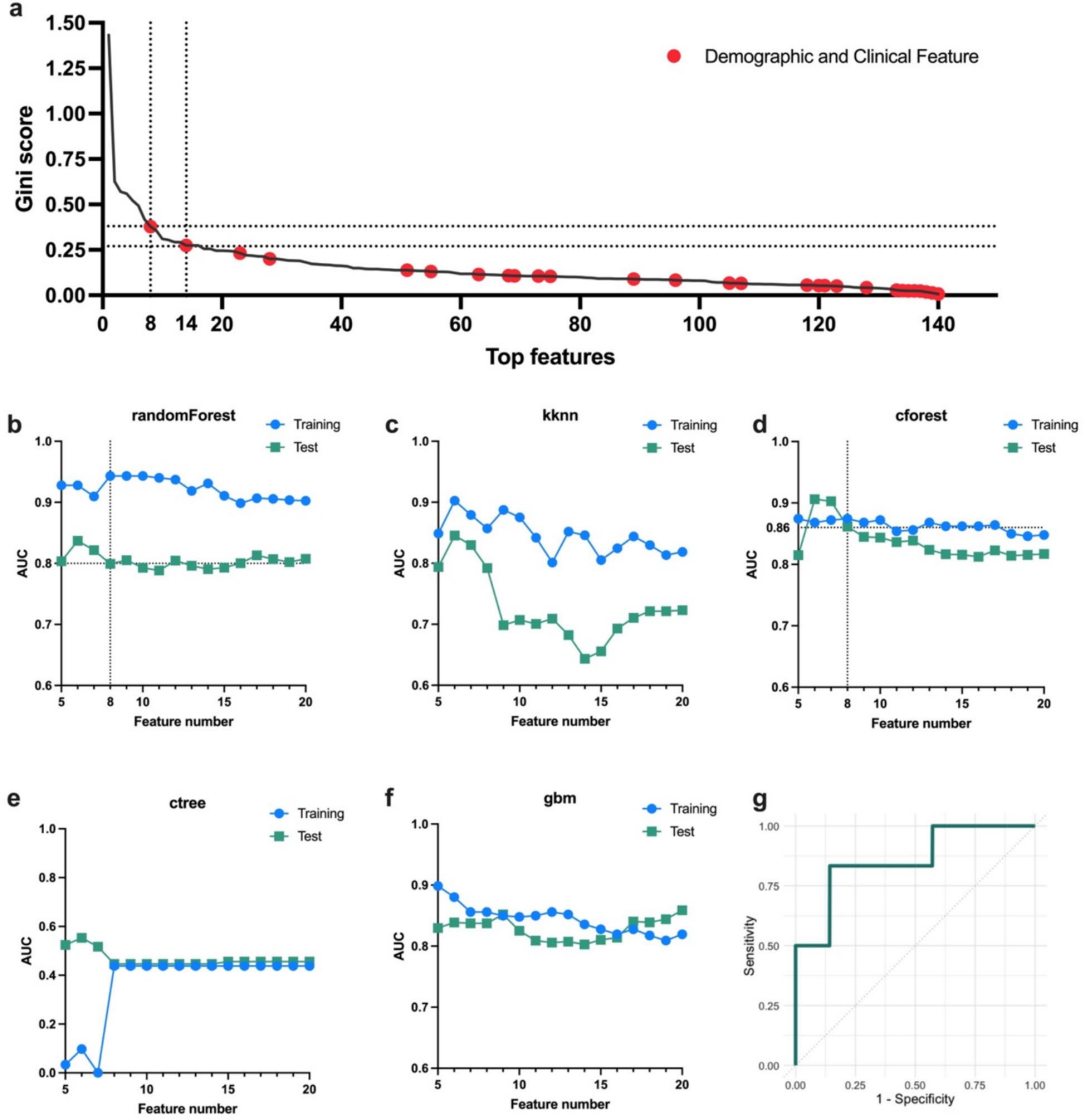
Integration of demographic, clinical, and CT data for multiomic model. **a** Features from pre-evacuation CT data were combined with demographic and clinical data, assigned Gini scores, and ranked in descending order. Demographic and clinical features are marked in red, with Post.RPRFX and ICP ranked 8th and 14th, respectively. **b** In the randomForest model, the average AUC in the test cohort stabilized around 0.8 when the feature set included more than 7 features. **d** In the cforest model, performance improved with more than 7 features but decreased as the feature set grew. Specifically, using the top 8 features achieved an AUC of 0.87 in the training cohort and 0.86 in the test cohort, with balanced sensitivity of 0.83 and specificity of 0.86 (**g**). **f** In the gbm model, the inclusion of Post.RPRFX led to a decline in AUC, which then stabilized around 0.8 with the top 15 features. **c**, **e** These models did not demonstrate satisfactory performance. AUC, area under the curve

To construct the multiomic model, we integrated features from pre-evacuation CT data with demographic and clinical data. All combined features were assigned Gini scores and ranked in descending order, as shown in Supplementary Table 3 and Fig. 4a. Among the top 20 features, Post.RPRFX and ICP ranked 8th and 14th, respectively, while the remaining features were radiomic features. In the randomForest model (Fig. 4b), when the feature set exceeded 8 features, the average AUC in the test cohort stabilized around 0.8, indicating no significant improvement with the inclusion of demographic and clinical data compared to the radiomic-only model (*p* = 0.73, *t*-test). In the cforest model (Fig. 4d), the performance became more robust with more than 7 features, but the AUC in the test cohort decreased as the number of features increased. Specifically, using the top 8 features yielded an AUC of 0.87 in the training cohort and 0.86 in the test cohort, with balanced sensitivity of 0.83 and specificity of 0.86 (Fig. 4g). In the gbm model (Fig. 4f), the inclusion of Post.RPRFX led to a decline in AUC in the test cohort, which stabilized around 0.8 with the top 15 features. The ACC performance metrics for these models can be found in Supplementary Fig. 4.

## Discussion

This study aimed to develop a predictive model to assess the likelihood of progressively refractory intracranial hypertension leading to secondary DC in TBI patients. Our results showed that the model based solely on demographic and clinical data did not yield satisfactory performance. In contrast, a robust predictive model was developed using only radiomic features extracted from the ROI of the lesions, achieving an AUC of 0.83 in the test cohort. When these radiomic features were combined with demographic and clinical data to form a multiomic model, the predictive performance improved, reaching an AUC of 0.86 in the test cohort. These predictive models have the potential to identify patients at risk of progressively refractory intracranial hypertension, enabling early intervention to prevent further deterioration.

The decision to replace the bone flap during hematoma evacuation remains a topic of debate among surgeons [25, 26]. Some advocate for primary DC, believing it effectively reduces intracranial pressure and lowers the risk of later deterioration. Others prefer bone flap replacement whenever possible, especially when continuous ICP monitoring can help assess sustained ICP elevation. Previous studies have provided limited guidance on this issue, and uncertainties persist regarding the optimal timing of secondary DC [27, 28]. Many studies combined both primary and secondary DC cases to address disparities in their respective outcomes [29]. We consider this combination inappropriate due to inherent selection bias. Patients undergoing primary DC frequently display clinical characteristics indicative of more severe TBI [30, 31], including lower GCS scores, larger hematoma volumes, substantial midline shifts, and pronounced extracranial injuries. To address this issue, our study specifically focused on secondary DC. We selectively enrolled patients who underwent craniotomy for hematoma evacuation with bone flap replacement, while excluding primary DC cases. This approach aimed to minimize selection bias and ensure a more standardized assessment of TBI severity across the study cohort.

The 2020 update of the Brain Trauma Foundation Guidelines for severe TBI suggests that late DC is preferable to early DC [3], citing potential improvements in mortality rates and clinical outcomes. This reinforces the idea that DC should be a reserve therapeutic option rather than a first-line intervention for managing intracranial hypertension [32]. Given this, patients undergoing hematoma evacuation for TBI require close monitoring for progressively refractory intracranial hypertension that may necessitate secondary DC. However, current clinical monitoring methods are limited by their reactive nature, as sustained ICP elevation may indicate that deterioration has begun. A robust predictive model capable of identifying high-risk patients in advance could enable earlier intervention and improve outcomes—this is the primary aim of our study.

Radiomics is a novel field that involves extracting quantitative data from medical images to assess the heterogeneity of lesions and explore their relationships with valuable clinical information [10, 33–36]. However, the application of radiomics and machine learning in TBI research is limited, with only a handful of studies available. Zhang et al. developed a radiomics-based model integrating radiomic data with clinical features to predict hematoma progression and clinical outcomes in TBI, demonstrating notable predictive performance for hematoma progression [11]. Pease et al. constructed a deep learning model utilizing head CT and clinical data, facilitating the prediction of 6-month outcomes following severe TBI [14]. Zheng et al. introduced a predictive nomogram that incorporates CT-based radiomic signatures and clinical features, demonstrating superior accuracy in the early prediction of in-hospital mortality [13]. These findings suggest that a radiomics-based model has the potential to significantly enhance predictive efficiency in the field of TBI. Both our radiomics-only and multiomic models demonstrated satisfactory performance, making this the first reported model applied to TBI patients.

Based on the top-ranking radiomic features in the radiomics-only model, we sought to interpret the model’s construction. Supplementary Table 2 reveals that the highest-ranked feature, Maskoriginal_VolumeNum, a diagnostic feature describing the ROI volume, had a Gini score significantly higher than all other features, underscoring its importance in the model. Among the other top 20 features, six belonged to the first-order subgroup, primarily characterizing the distribution of voxel intensities within the ROI. Four features were from the GLSZM subgroup, quantifying gray level zones within the ROI. Three were shape-related features that measured the size and geometric characteristics of the ROI. Two features were from the GLDM, quantifying gray level dependencies, while another two were from the GLCM, describing the second-order joint probability function of an image region constrained by the mask. Lastly, two features were from the NGTDM, quantifying the difference between a gray value and the average gray value of its neighbors. These radiomic features capture detailed information about lesion shape and texture and have been shown to strongly correlate with underlying pathobiological changes influencing the macroscopic appearance of pixel intensity [37–39]. Using machine learning, the most relevant features were selected to construct the predictive model, which demonstrated satisfactory performance. These findings suggest that radiomic features may serve as imaging biomarkers, uncovering deep imaging characteristics that conventional radiographic interpretation methods cannot readily detect. Additionally, when comparing the top radiomic features in the multiomic model, 13 out of 18 overlapped, further demonstrating the robustness of the most relevant features used in the models.

The development of predictive models presents several challenges and limitations. First, the patient cohort size was relatively small, and the limited number of outcome events in the training set reduced the statistical power and may limit the generalizability of our model. This limitation primarily reflects the relatively low incidence of secondary DC within the study period and the single-center nature of our dataset. Second, manual segmentation of the ROIs was performed by experienced neurosurgeons. Although this ensured accurate delineation, the process is time-consuming and subject to inter- and intra-observer variability, which may affect reproducibility. Third, the proposed workflow—involving manual segmentation, radiomics extraction, and machine learning—is inherently complex. While applicable in clinical practice, it may limit rapid implementation in ICU or emergency settings. Future work should focus on incorporating semi-automated or fully automated segmentation methods and integrating the pipeline into clinical workflows to enhance practical applicability. In addition, expanding multicenter datasets, refining and validating models, and addressing variability and heterogeneity in pre-evacuation imaging data will be important.

## Conclusion

In this study, we developed radiomics-based predictive models to estimate the risk of progressively refractory intracranial hypertension requiring secondary DC in a selected cohort of TBI patients who had already undergone emergent craniotomy for hematoma evacuation with bone flap replacement. The model relying solely on radiomic features extracted from the lesion demonstrated satisfactory performance. When these features were integrated with demographic and clinical data to create a multiomic model, predictive performance further improved. These findings highlight the model’s potential to identify high-risk patients, enabling early intervention to prevent further deterioration.

## Supplementary Information

Supplementary Material 1.

Supplementary Material 2.

Supplementary Material 3.

Supplementary Material 4.

Supplementary Material 5.

Supplementary Material 6.

Supplementary Material 7.

## Data Availability

The radiomic model and analytical code used for the analyses are accessible on GitHub (https://github.com/DrSunZY/Secondary-DC). De-identified data from this study will be made available in accordance with institutional IRB standards, accessible by contacting the corresponding author.

## Abbreviations

ACC: Accuracy
ALB: Albumin
AUC: Area under the curve
CT: Computed tomography
DC: Decompressive craniectomy
FDR: False discovery rate
GCS: Glasgow Coma Scale
GLCM: Gray Level Co-occurrence Matrix
GLDM: Gray Level Dependence Matrix
GLRLM: Gray Level Run Length Matrix
GLSZM: Gray Level Size Zone Matrix
HB: Hemoglobin
ICP: Intracranial pressure
LOO-CV: Leave-One-Out Cross-Validation
MDI: Mean decrease in impurity
NDC group: Non-Decompressive Craniectomy Group
NGTDM: Neighboring Gray Tone Difference Matrix
PRFXscore: Pupil Light Reflex Score
ROI: Region of interest
TBI: Traumatic brain injury
WBC: White blood cell count
