# Supplementary material for: Radiomics-based machine learning model for predicting secondary decompressive craniectomy in TBI patients after emergent craniotomy with bone flap replacement
Source: Chin Neurosurg J. 2026 Jan 8;12:1. doi: 10.1186/s41016-025-00423-5 (PMC12781376; doi:10.1186/s41016-025-00423-5)
Supplement: Supplementary file 2 — Supplementary Material 2. [file 41016_2025_423_MOESM2_ESM.docx]

**Supplementary Table 2.** Radiomic Features from Pre-evacuation CT with Gini Scores (Descending Order)

| **Order** | **Radiomic Feature** | **Score** |
| --- | --- | --- |
| 1 | diagnostics_Maskoriginal_VolumeNum | 1.85 |
| 2 | original_firstorder_Maximum | 0.74 |
| 3 | original_glszm_GrayLevelVariance | 0.62 |
| 4 | original_ngtdm_Strength | 0.57 |
| 5 | original_firstorder_Skewness | 0.52 |
| 6 | original_shape_LeastAxisLength | 0.47 |
| 7 | original_gldm_DependenceNonUniformity | 0.46 |
| 8 | original_glszm_ZoneEntropy | 0.46 |
| 9 | original_firstorder_Kurtosis | 0.43 |
| 10 | original_ngtdm_Busyness | 0.38 |
| 11 | original_shape_Maximum2DDiameterRow | 0.37 |
| 12 | original_glszm_GrayLevelNonUniformity | 0.37 |
| 13 | original_glcm_Idmn | 0.36 |
| 14 | original_firstorder_Energy | 0.32 |
| 15 | original_firstorder_Uniformity | 0.30 |
| 16 | original_firstorder_Range | 0.30 |
| 17 | original_glszm_ZoneVariance | 0.29 |
| 18 | original_gldm_DependenceVariance | 0.28 |
| 19 | original_glcm_Idn | 0.27 |
| 20 | original_shape_Flatness | 0.27 |
| 21 | original_glcm_MCC | 0.25 |
| 22 | original_ngtdm_Coarseness | 0.25 |
| 23 | original_glszm_GrayLevelNonUniformityNormalized | 0.24 |
| 24 | original_ngtdm_Complexity | 0.24 |
| 25 | original_gldm_GrayLevelVariance | 0.24 |
| 26 | original_firstorder_TotalEnergy | 0.23 |
| 27 | original_shape_SurfaceArea | 0.23 |
| 28 | original_glszm_LargeAreaEmphasis | 0.22 |
| 29 | original_ngtdm_Contrast | 0.20 |
| 30 | original_firstorder_MeanAbsoluteDeviation | 0.18 |
| 31 | original_glcm_ClusterShade | 0.18 |
| 32 | original_shape_MeshVolume | 0.18 |
| 33 | original_glszm_SmallAreaEmphasis | 0.18 |
| 34 | original_glrlm_RunVariance | 0.17 |
| 35 | original_glszm_SizeZoneNonUniformity | 0.17 |
| 36 | original_shape_Sphericity | 0.17 |
| 37 | original_glrlm_ShortRunLowGrayLevelEmphasis | 0.17 |
| 38 | original_shape_Maximum2DDiameterSlice | 0.16 |
| 39 | original_firstorder_Minimum | 0.16 |
| 40 | original_firstorder_Median | 0.16 |
| 41 | original_glrlm_LongRunEmphasis | 0.16 |
| 42 | original_shape_Maximum2DDiameterColumn | 0.16 |
| 43 | original_firstorder_RootMeanSquared | 0.15 |
| 44 | original_shape_MinorAxisLength | 0.15 |
| 45 | original_glcm_InverseVariance | 0.14 |
| 46 | original_firstorder_90Percentile | 0.14 |
| 47 | original_glrlm_GrayLevelVariance | 0.14 |
| 48 | original_gldm_SmallDependenceLowGrayLevelEmphasis | 0.14 |
| 49 | original_gldm_DependenceEntropy | 0.14 |
| 50 | diagnostics_Maskoriginal_VoxelNum | 0.14 |
| 51 | original_glcm_Imc1 | 0.13 |
| 52 | original_glcm_JointEntropy | 0.13 |
| 53 | original_glcm_ClusterProminence | 0.13 |
| 54 | original_firstorder_Entropy | 0.13 |
| 55 | diagnostics_Imageoriginal_Mean | 0.13 |
| 56 | original_glrlm_GrayLevelNonUniformityNormalized | 0.13 |
| 57 | original_gldm_LargeDependenceLowGrayLevelEmphasis | 0.13 |
| 58 | original_firstorder_Mean | 0.13 |
| 59 | original_glszm_LargeAreaHighGrayLevelEmphasis | 0.13 |
| 60 | diagnostics_Imageoriginal_Maximum | 0.13 |
| 61 | original_glrlm_RunPercentage | 0.13 |
| 62 | original_glszm_ZonePercentage | 0.13 |
| 63 | original_shape_VoxelVolume | 0.13 |
| 64 | original_glcm_ClusterTendency | 0.12 |
| 65 | original_shape_MajorAxisLength | 0.12 |
| 66 | original_glcm_SumSquares | 0.12 |
| 67 | original_glszm_SizeZoneNonUniformityNormalized | 0.12 |
| 68 | original_gldm_DependenceNonUniformityNormalized | 0.12 |
| 69 | original_firstorder_Variance | 0.12 |
| 70 | original_glrlm_LongRunHighGrayLevelEmphasis | 0.11 |
| 71 | original_glcm_Imc2 | 0.11 |
| 72 | original_glcm_DifferenceAverage | 0.11 |
| 73 | original_glrlm_RunLengthNonUniformity | 0.11 |
| 74 | original_glcm_Contrast | 0.11 |
| 75 | original_firstorder_10Percentile | 0.11 |
| 76 | original_shape_SurfaceVolumeRatio | 0.11 |
| 77 | original_glrlm_HighGrayLevelRunEmphasis | 0.11 |
| 78 | original_shape_Maximum3DDiameter | 0.11 |
| 79 | original_glrlm_RunEntropy | 0.10 |
| 80 | original_glszm_LargeAreaLowGrayLevelEmphasis | 0.10 |
| 81 | original_glcm_MaximumProbability | 0.10 |
| 82 | original_firstorder_RobustMeanAbsoluteDeviation | 0.10 |
| 83 | original_glcm_DifferenceEntropy | 0.10 |
| 84 | original_glszm_SmallAreaHighGrayLevelEmphasis | 0.09 |
| 85 | original_glrlm_ShortRunEmphasis | 0.09 |
| 86 | original_glcm_SumEntropy | 0.09 |
| 87 | original_glcm_Correlation | 0.09 |
| 88 | original_gldm_GrayLevelNonUniformity | 0.09 |
| 89 | diagnostics_Imageoriginal_Minimum | 0.09 |
| 90 | original_glcm_Autocorrelation | 0.09 |
| 91 | original_gldm_SmallDependenceHighGrayLevelEmphasis | 0.08 |
| 92 | original_gldm_LowGrayLevelEmphasis | 0.08 |
| 93 | original_glcm_JointEnergy | 0.08 |
| 94 | original_glcm_Id | 0.08 |
| 95 | original_glrlm_RunLengthNonUniformityNormalized | 0.07 |
| 96 | original_glrlm_LongRunLowGrayLevelEmphasis | 0.07 |
| 97 | original_firstorder_InterquartileRange | 0.07 |
| 98 | original_gldm_LargeDependenceHighGrayLevelEmphasis | 0.07 |
| 99 | original_glszm_HighGrayLevelZoneEmphasis | 0.07 |
| 100 | original_gldm_HighGrayLevelEmphasis | 0.07 |
| 101 | original_shape_Elongation | 0.06 |
| 102 | original_glcm_SumAverage | 0.06 |
| 103 | original_glszm_LowGrayLevelZoneEmphasis | 0.06 |
| 104 | original_glcm_DifferenceVariance | 0.06 |
| 105 | original_glrlm_ShortRunHighGrayLevelEmphasis | 0.05 |
| 106 | original_glszm_SmallAreaLowGrayLevelEmphasis | 0.05 |
| 107 | original_glcm_Idm | 0.05 |
| 108 | original_glrlm_GrayLevelNonUniformity | 0.05 |
| 109 | original_gldm_SmallDependenceEmphasis | 0.05 |
| 110 | original_gldm_LargeDependenceEmphasis | 0.04 |
| 111 | original_glcm_JointAverage | 0.04 |
| 112 | original_glrlm_LowGrayLevelRunEmphasis | 0.04 |

Radiomic features consist of 5 diagnostic features and 107 original features, further categorized into seven groups: shape-based features, first-order statistics, gray level co-occurrence matrix (GLCM) features, gray level dependence matrix (GLDM) features, gray level run length matrix (GLRLM) features, gray level size zone matrix (GLSZM) features, and neighboring gray tone difference matrix (NGTDM) features.
