# Supplementary material for: Radiomics-based machine learning model for predicting secondary decompressive craniectomy in TBI patients after emergent craniotomy with bone flap replacement
Source: Chin Neurosurg J. 2026 Jan 8;12:1. doi: 10.1186/s41016-025-00423-5 (PMC12781376; doi:10.1186/s41016-025-00423-5)
Supplement: Supplementary file 3 — Supplementary Material 3. [file 41016_2025_423_MOESM3_ESM.docx]

**Supplementary Table 3.** Radiomic, Demographic, and Clinical Features

with Gini Scores (Descending Order)

| **Order** | **Radiomic, Demographic, and Clinical Feature** | **Score** |
| --- | --- | --- |
| 1 | diagnostics_Maskoriginal_VolumeNum | 1.43336 |
| 2 | original_ngtdm_Strength | 0.62737 |
| 3 | original_glszm_GrayLevelVariance | 0.571068 |
| 4 | original_firstorder_Maximum | 0.55939 |
| 5 | original_gldm_DependenceNonUniformity | 0.521385 |
| 6 | original_shape_LeastAxisLength | 0.493324 |
| 7 | original_firstorder_Skewness | 0.419072 |
| 8 | Post.RPRFX | 0.37803 |
| 9 | original_glszm_ZoneEntropy | 0.355179 |
| 10 | original_glszm_GrayLevelNonUniformity | 0.310707 |
| 11 | original_shape_Maximum2DDiameterRow | 0.303851 |
| 12 | original_glszm_ZoneVariance | 0.292879 |
| 13 | original_ngtdm_Contrast | 0.291344 |
| 14 | ICP | 0.27481 |
| 15 | original_firstorder_Kurtosis | 0.274392 |
| 16 | original_firstorder_TotalEnergy | 0.274001 |
| 17 | original_glcm_MCC | 0.255461 |
| 18 | original_glcm_Idmn | 0.255269 |
| 19 | original_ngtdm_Complexity | 0.246062 |
| 20 | original_shape_SurfaceArea | 0.245638 |
| 21 | original_firstorder_Range | 0.244258 |
| 22 | original_glszm_GrayLevelNonUniformityNormalized | 0.242274 |
| 23 | AGE | 0.232196 |
| 24 | original_glcm_Idn | 0.22024 |
| 25 | original_firstorder_Energy | 0.218643 |
| 26 | original_ngtdm_Busyness | 0.213799 |
| 27 | original_firstorder_Entropy | 0.212635 |
| 28 | Adm.GCS | 0.200611 |
| 29 | original_glcm_ClusterShade | 0.200516 |
| 30 | original_shape_Flatness | 0.197225 |
| 31 | original_glszm_SizeZoneNonUniformity | 0.191256 |
| 32 | original_firstorder_Uniformity | 0.19054 |
| 33 | original_firstorder_MeanAbsoluteDeviation | 0.188937 |
| 34 | original_shape_Maximum2DDiameterSlice | 0.181835 |
| 35 | original_glrlm_GrayLevelVariance | 0.173345 |
| 36 | original_gldm_GrayLevelNonUniformity | 0.170287 |
| 37 | diagnostics_Maskoriginal_VoxelNum | 0.1684 |
| 38 | diagnostics_Imageoriginal_Mean | 0.166929 |
| 39 | original_firstorder_RobustMeanAbsoluteDeviation | 0.162693 |
| 40 | original_glszm_LargeAreaHighGrayLevelEmphasis | 0.16207 |
| 41 | original_glszm_LargeAreaEmphasis | 0.159245 |
| 42 | original_firstorder_Mean | 0.149743 |
| 43 | original_glrlm_RunVariance | 0.148833 |
| 44 | original_glrlm_RunPercentage | 0.145947 |
| 45 | original_shape_MajorAxisLength | 0.144761 |
| 46 | original_glcm_MaximumProbability | 0.14383 |
| 47 | original_shape_Maximum2DDiameterColumn | 0.142361 |
| 48 | original_glrlm_GrayLevelNonUniformityNormalized | 0.140225 |
| 49 | original_firstorder_Median | 0.138155 |
| 50 | original_gldm_DependenceVariance | 0.138042 |
| 51 | PLT | 0.137517 |
| 52 | original_shape_Elongation | 0.136074 |
| 53 | original_shape_VoxelVolume | 0.134182 |
| 54 | original_glcm_ClusterTendency | 0.134152 |
| 55 | INR | 0.130166 |
| 56 | original_glcm_SumSquares | 0.129496 |
| 57 | original_glszm_SizeZoneNonUniformityNormalized | 0.129013 |
| 58 | original_glcm_ClusterProminence | 0.128698 |
| 59 | original_gldm_GrayLevelVariance | 0.123127 |
| 60 | original_glszm_SmallAreaHighGrayLevelEmphasis | 0.118275 |
| 61 | original_glcm_Imc1 | 0.117993 |
| 62 | original_gldm_SmallDependenceHighGrayLevelEmphasis | 0.11781 |
| 63 | Post.GCS | 0.114484 |
| 64 | original_glcm_InverseVariance | 0.114457 |
| 65 | original_gldm_LargeDependenceEmphasis | 0.112674 |
| 66 | original_gldm_LargeDependenceHighGrayLevelEmphasis | 0.111943 |
| 67 | original_shape_MinorAxisLength | 0.110282 |
| 68 | FBG | 0.108058 |
| 69 | APTT | 0.107391 |
| 70 | original_glcm_DifferenceAverage | 0.106363 |
| 71 | original_gldm_SmallDependenceLowGrayLevelEmphasis | 0.106227 |
| 72 | original_glszm_LargeAreaLowGrayLevelEmphasis | 0.106061 |
| 73 | HB | 0.10599 |
| 74 | original_glszm_ZonePercentage | 0.1052 |
| 75 | Post.LPRFX | 0.10489 |
| 76 | original_gldm_LargeDependenceLowGrayLevelEmphasis | 0.103021 |
| 77 | original_shape_MeshVolume | 0.102171 |
| 78 | original_ngtdm_Coarseness | 0.101002 |
| 79 | original_shape_Maximum3DDiameter | 0.100598 |
| 80 | original_glcm_Imc2 | 0.098893 |
| 81 | original_glcm_Id | 0.096749 |
| 82 | original_glrlm_LongRunEmphasis | 0.095027 |
| 83 | original_glrlm_LongRunHighGrayLevelEmphasis | 0.092146 |
| 84 | original_glrlm_ShortRunEmphasis | 0.091779 |
| 85 | original_shape_Sphericity | 0.091638 |
| 86 | original_glszm_SmallAreaEmphasis | 0.091017 |
| 87 | original_gldm_DependenceNonUniformityNormalized | 0.090491 |
| 88 | original_glcm_SumEntropy | 0.090383 |
| 89 | Adm.RPD | 0.089602 |
| 90 | original_glrlm_RunLengthNonUniformityNormalized | 0.087513 |
| 91 | original_glcm_Contrast | 0.087462 |
| 92 | original_glcm_DifferenceVariance | 0.087271 |
| 93 | original_shape_SurfaceVolumeRatio | 0.086995 |
| 94 | original_glcm_JointEnergy | 0.085787 |
| 95 | original_glszm_HighGrayLevelZoneEmphasis | 0.085303 |
| 96 | NA | 0.08286 |
| 97 | original_firstorder_Minimum | 0.081895 |
| 98 | original_firstorder_RootMeanSquared | 0.081867 |
| 99 | original_glrlm_HighGrayLevelRunEmphasis | 0.080566 |
| 100 | original_glrlm_ShortRunLowGrayLevelEmphasis | 0.080226 |
| 101 | original_firstorder_10Percentile | 0.078853 |
| 102 | original_glcm_JointEntropy | 0.072912 |
| 103 | original_glcm_Idm | 0.071225 |
| 104 | original_firstorder_90Percentile | 0.070923 |
| 105 | ALB | 0.066784 |
| 106 | original_glcm_JointAverage | 0.066715 |
| 107 | NEUT | 0.066099 |
| 108 | original_firstorder_Variance | 0.06259 |
| 109 | original_glszm_SmallAreaLowGrayLevelEmphasis | 0.0618 |
| 110 | original_glrlm_GrayLevelNonUniformity | 0.061405 |
| 111 | original_glrlm_ShortRunHighGrayLevelEmphasis | 0.061197 |
| 112 | original_gldm_DependenceEntropy | 0.060044 |
| 113 | original_gldm_SmallDependenceEmphasis | 0.059704 |
| 114 | original_glrlm_RunLengthNonUniformity | 0.057708 |
| 115 | original_glrlm_RunEntropy | 0.056757 |
| 116 | original_firstorder_InterquartileRange | 0.056617 |
| 117 | diagnostics_Imageoriginal_Maximum | 0.056394 |
| 118 | WBC | 0.055785 |
| 119 | original_glrlm_LongRunLowGrayLevelEmphasis | 0.0557 |
| 120 | CL | 0.053089 |
| 121 | LY | 0.052969 |
| 122 | original_glszm_LowGrayLevelZoneEmphasis | 0.051894 |
| 123 | K | 0.050643 |
| 124 | original_glcm_Autocorrelation | 0.047544 |
| 125 | original_glrlm_LowGrayLevelRunEmphasis | 0.046759 |
| 126 | diagnostics_Imageoriginal_Minimum | 0.043482 |
| 127 | original_gldm_HighGrayLevelEmphasis | 0.042222 |
| 128 | PT | 0.041268 |
| 129 | original_glcm_Correlation | 0.041185 |
| 130 | original_gldm_LowGrayLevelEmphasis | 0.039756 |
| 131 | original_glcm_DifferenceEntropy | 0.036364 |
| 132 | original_glcm_SumAverage | 0.034063 |
| 133 | Post.RPD | 0.027998 |
| 134 | SEX | 0.025446 |
| 135 | Post.LPD | 0.024689 |
| 136 | Rotterdam score | 0.024081 |
| 137 | Adm.RPRFX | 0.022557 |
| 138 | MOI | 0.018476 |
| 139 | Adm.LPD | 0.013387 |
| 140 | Adm.LPRFX | 0.007443 |

Demographic and clinical features are indicated in red font. Radiomic features consist of 5 diagnostic features and 107 original features, further categorized into seven groups: shape-based features, first-order statistics, gray level co-occurrence matrix (GLCM) features, gray level dependence matrix (GLDM) features, gray level run length matrix (GLRLM) features, gray level size zone matrix (GLSZM) features, and neighboring gray tone difference matrix (NGTDM) features. Adm., upon administration to the hospital; LPD, left pupil diameter; LPRFX, left pupil light reflex; RPD, right pupil diameter; RPRFX, right pupil light reflex; GCS, Glasgow Coma Scale; ICP, intracranial pressure; Post., post emergent evacuation procedure within 8 hours; HB, hemoglobin; WBC, white blood cell; NEUT, neutrophils; LY, lymphocytes; PLT, platelet; PT, prothrombin time; APTT, activated partial thromboplastin time; INR, international normalized ratio; FBG, fasting blood glucose; ALB, albumin; K, potassium; NA, sodium; CL, chloride; MOI, Mechanisms of injury.
