# Supplementary figures and images for: Radiomics-based machine learning model for predicting secondary decompressive craniectomy in TBI patients after emergent craniotomy with bone flap replacement
Source: Chin Neurosurg J. 2026 Jan 8;12:1. doi: 10.1186/s41016-025-00423-5 (PMC12781376; doi:10.1186/s41016-025-00423-5)

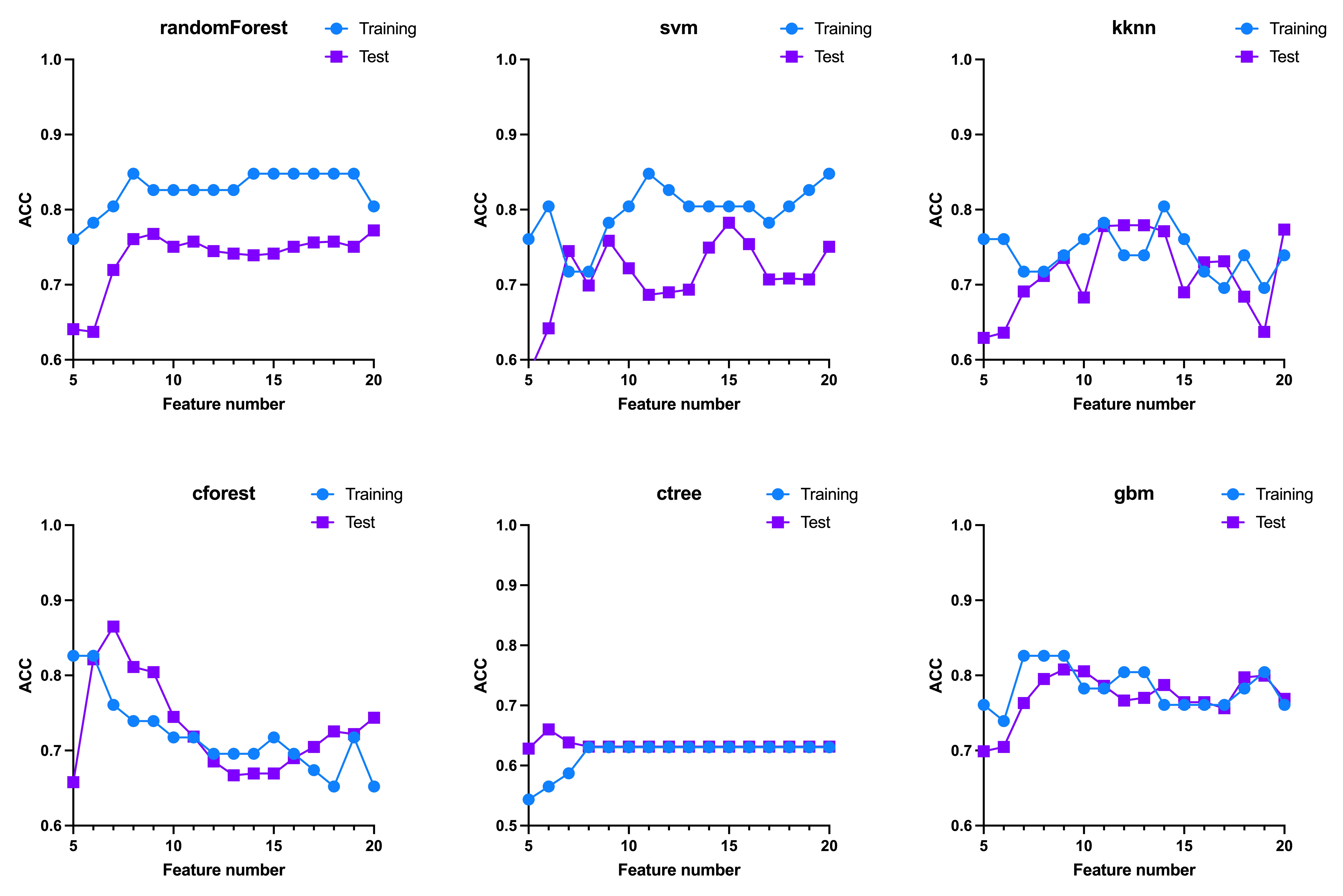

Supplement: Supplementary file 4 — Supplementary Material 4. [file 41016_2025_423_MOESM4_ESM.jpg]

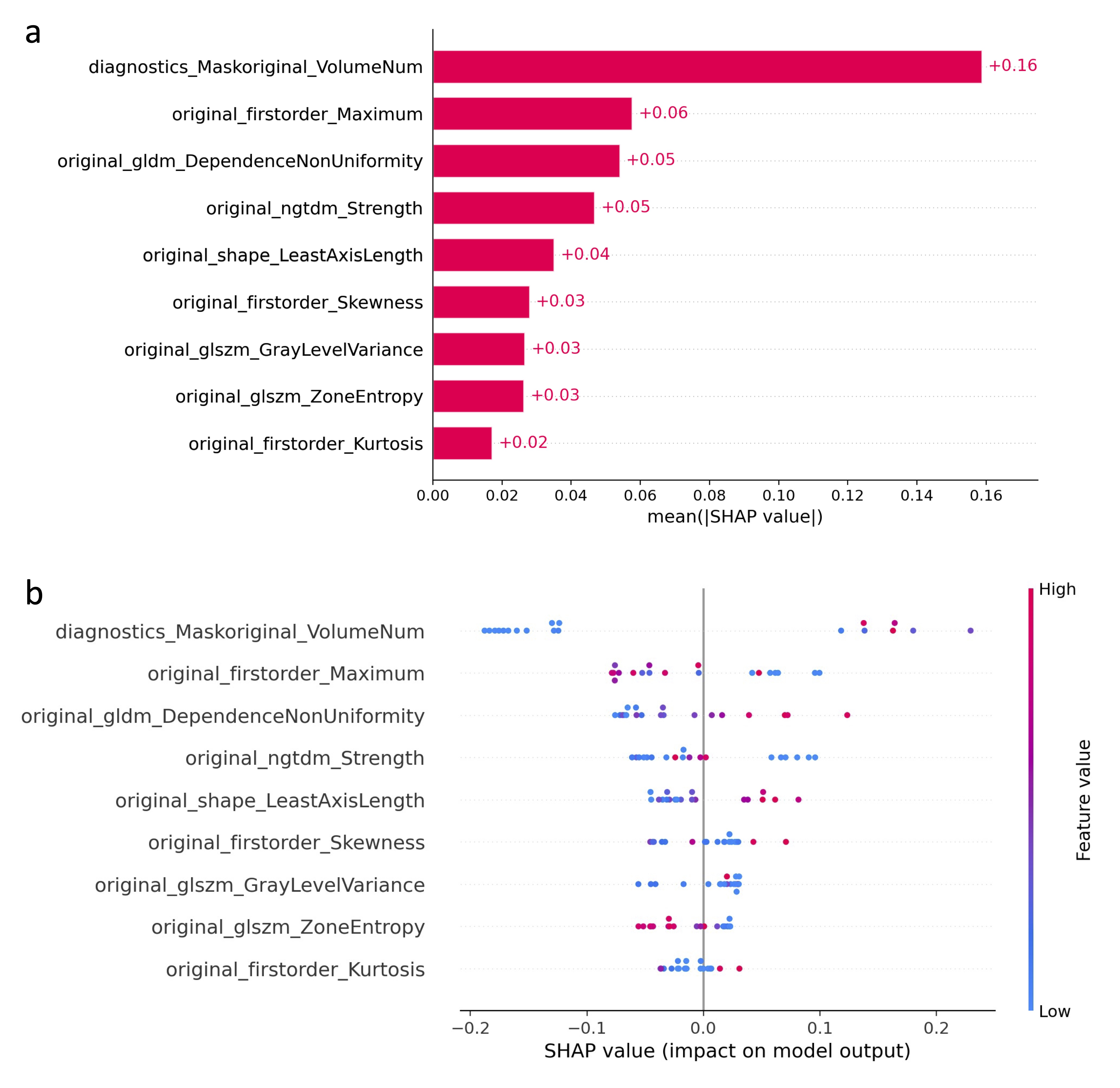

Supplement: Supplementary file 5 — Supplementary Material 5. [file 41016_2025_423_MOESM5_ESM.jpg]

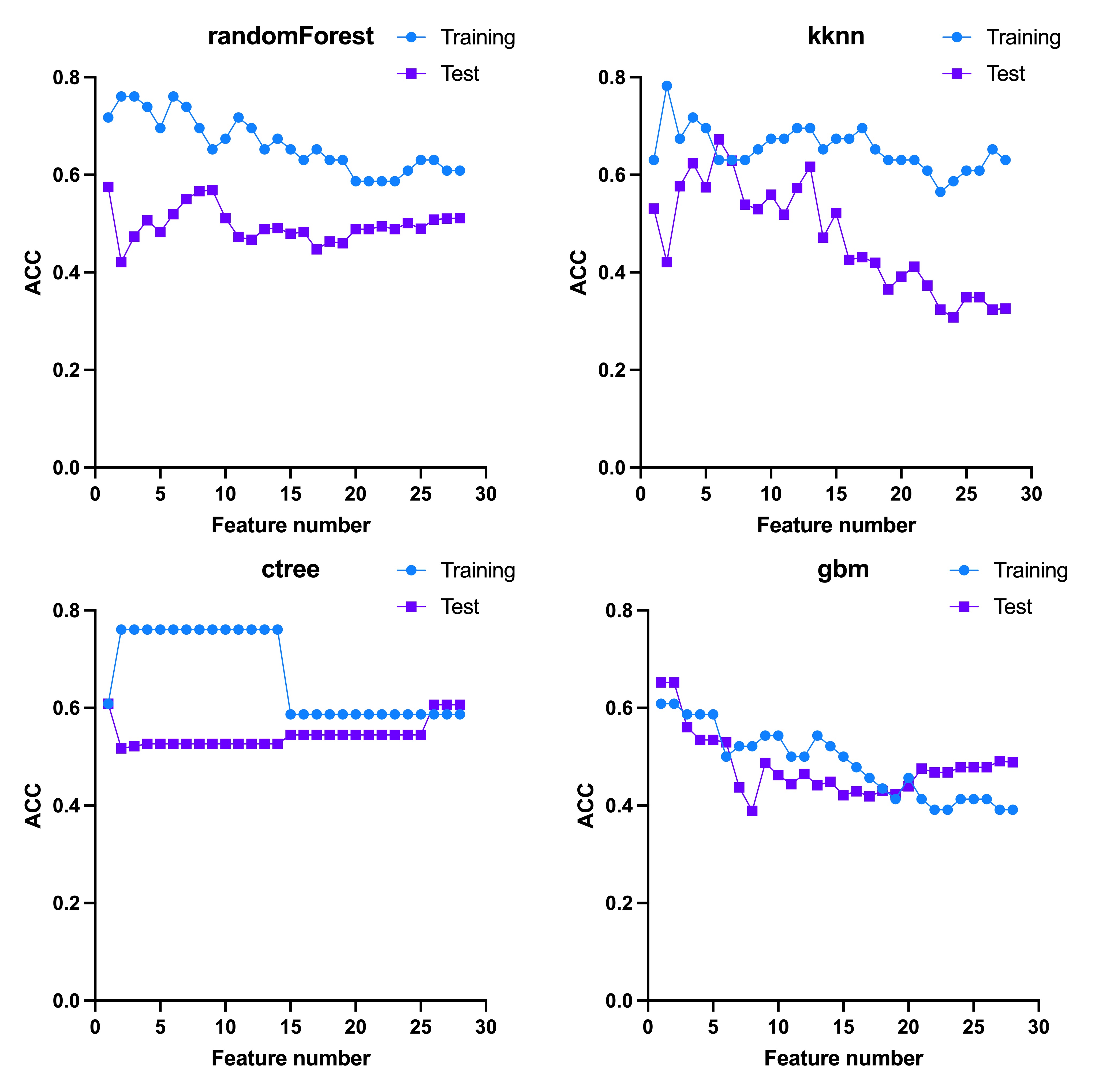

Supplement: Supplementary file 6 — Supplementary Material 6. [file 41016_2025_423_MOESM6_ESM.jpg]

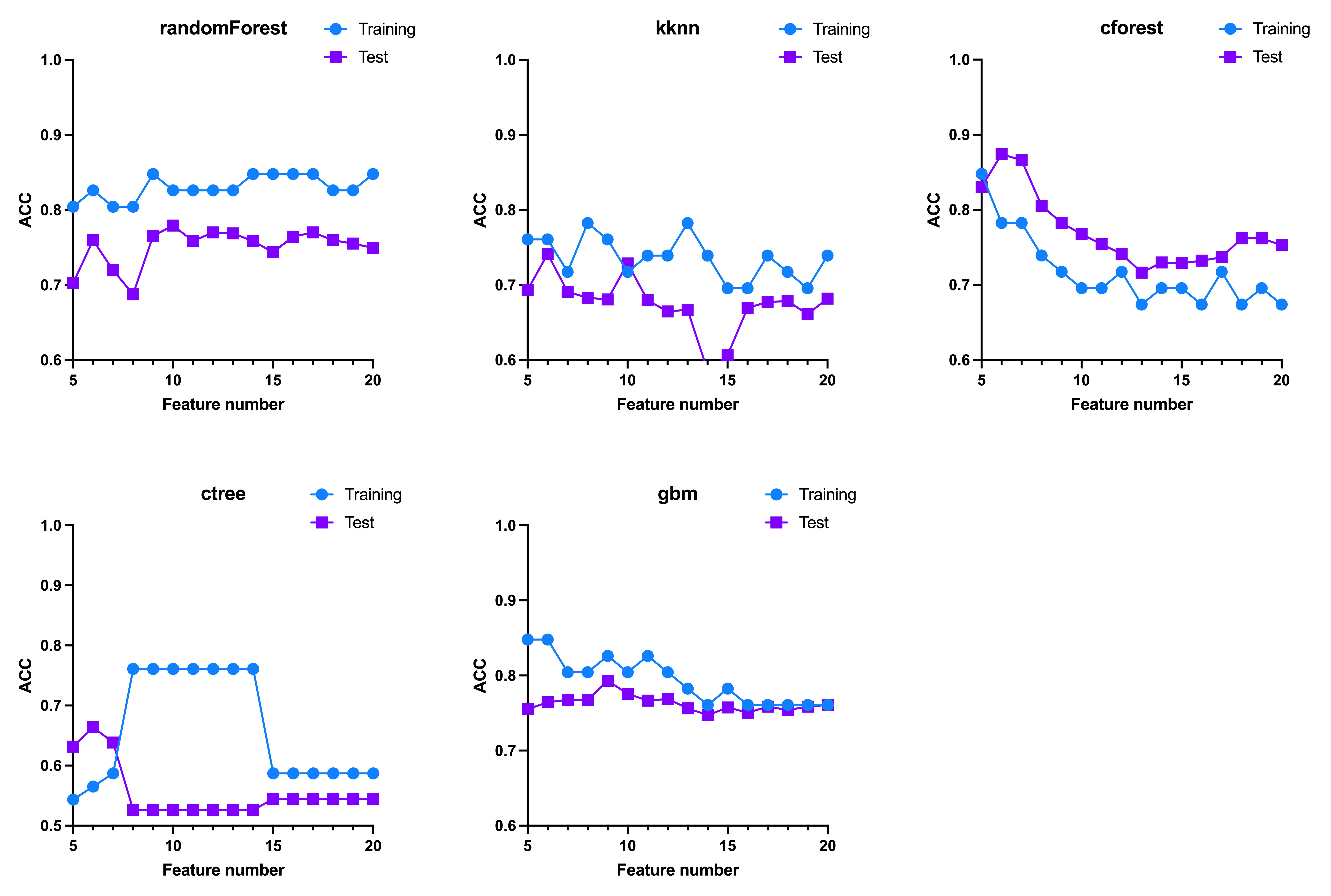

Supplement: Supplementary file 7 — Supplementary Material 7. [file 41016_2025_423_MOESM7_ESM.jpg]
